# Chitosan Based Self-Assembled Nanoparticles in Drug Delivery

**DOI:** 10.3390/polym10030235

**Published:** 2018-02-26

**Authors:** Javier Pérez Quiñones, Hazel Peniche, Carlos Peniche

**Affiliations:** 1 Institute of Polymer Chemistry, Johannes Kepler University, Altenberger Strasse 69, 4040 Linz, Austria; javenator@gmail.com; 2 Centro de Biomateriales, Universidad de La Habana, Ave. Universidad S/N entre G y Ronda, 10400 La Habana, Cuba; hazel@biomat.uh.cu; 3 Facultad de Química, Universidad de La Habana, Zapata S/N entre G y Carlitos Aguirre, 10400 La Habana, Cuba

**Keywords:** chitosan, self-assembled, polyelectrolyte complex, nanoparticle, drug delivery

## Abstract

Chitosan is a cationic polysaccharide that is usually obtained by alkaline deacetylation of chitin poly(*N*-acetylglucosamine). It is biocompatible, biodegradable, mucoadhesive, and non-toxic. These excellent biological properties make chitosan a good candidate for a platform in developing drug delivery systems having improved biodistribution, increased specificity and sensitivity, and reduced pharmacological toxicity. In particular, chitosan nanoparticles are found to be appropriate for non-invasive routes of drug administration: oral, nasal, pulmonary and ocular routes. These applications are facilitated by the absorption-enhancing effect of chitosan. Many procedures for obtaining chitosan nanoparticles have been proposed. Particularly, the introduction of hydrophobic moieties into chitosan molecules by grafting to generate a hydrophobic-hydrophilic balance promoting self-assembly is a current and appealing approach. The grafting agent can be a hydrophobic moiety forming micelles that can entrap lipophilic drugs or it can be the drug itself. Another suitable way to generate self-assembled chitosan nanoparticles is through the formation of polyelectrolyte complexes with polyanions. This paper reviews the main approaches for preparing chitosan nanoparticles by self-assembly through both procedures, and illustrates the state of the art of their application in drug delivery.

## 1. Introduction

**Figure 1:**
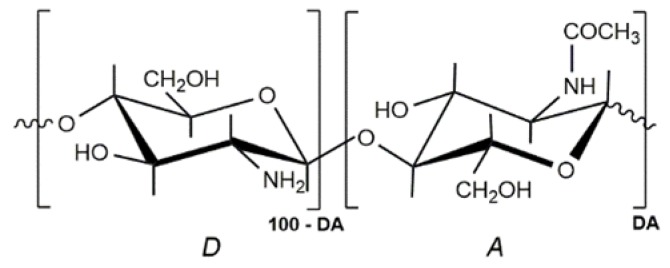
Structural units of chitin and chitosan. (A) *N*-acetylglucosamine unit; (D) Glucosamine unit. In chitosan DA < 50.

Chitosan (CS) is a family of linear polysaccharides that is composed of glucosamine and *N*-acetylglucosamine units linked together by β (1 → 4) glycosidic links (Figure 1). CS is obtained by the partial deacetylation of the naturally occurring polysaccharide, chitin, which is essentially poly(*N*-acetylglucosamine). Depending on the natural source and the conditions used to isolate and deacetylate chitin, the resulting degree of acetylation (DA) and molecular weight of chitosan will depend on the reaction parameters that are involved [1]. Molecular weight, the DA, and even the pattern of acetylation (the distribution of glucosamine and *N*-acetylglucosamine units along the chitosan chain) will affect its chemical and biological properties [2,3].

The degree of deacetylation (DD = 100 − DA) of chitosan is about 50% or higher. In dilute aqueous acid solutions, the amino groups of chitosan become protonated, allowing for its dissolution. In fact, the solubility of chitosan in 1% or 0.1 M acetic acid is a simple and practical criterion used to differentiate it from chitin. However, chitosan solubility depends on its DD, the ionic concentration, the pH, and the distribution of acetyl groups along the chain, as well as the conditions of isolation and drying. If deacetylation of chitin is performed under homogeneous conditions chitosans with a DD about 50% might dissolve, but if deacetylation is carried out under heterogeneous conditions, DD of 65% or higher is usually needed to achieve dissolution [4].

Chitosan is a biocompatible, biodegradable, and non-toxic material. It exhibits other significant biological properties, such as wound healing capacity, antimicrobial and hemostatic activities. It is an excellent film former and can be processed into fibers, gels, microspheres-microcapsules, and micro/nanoparticles [5]. Also, because it has free –OH and –NH_2_ groups in its structure, it is amenable to chemical modifications that can potentiate some of its properties for certain applications. All of these remarkable physical, chemical, and biological properties have made CS an excellent candidate for applications in cosmetics, food industry, medicine and pharmacy [4]. The preference of chitosan in comparison with other cationic polymers, such as polylysine, polyarginin, or polyethyleneimine for many of these applications relies on its comparatively lower toxicity [6].

Mucoadhesive and absorption-enhancing properties are also found in CS. It opens the tight junctions between cells so that the drug of interest can traverse the mucosal cells. [7,8]. These properties also make CS an ideal candidate for the delivery of drugs and bioactive molecules in general. Numerous reports show the applications of CS in drug delivery, with several reviews on the subject [6,8,9,10]. Applications include CS as an excipient in tablets, chitosan hydrogels, films, fibers, micro/nanocapsules and micro/nanoparticles.

Nanoparticles of CS are applied in drug delivery, not only by the traditional administration routes (e.g., oral and parenteral routes), but also via mucosal (nasal, pulmonary, vaginal) and ocular routes [11]. Chitosan nanoparticles are as well used in designing non-viral vectors for gene delivery and the delivery of vaccines [12].

Different approaches have been used to produce CS nanoparticles. These include ionotropic gelation [13,14], spray drying [15], water-in-oil emulsion cross-linking [16], reverse micelle formation [17,18], emulsion-droplet coalescence [19,20], nanoprecipitation [21], and by a self-assembling mechanism [22,23].

The self-assembling has been described as the association of certain molecules, macromolecules, or composite materials with themselves to form tridimensional networks or other structures with new distinguishing properties. The self-assembling process can take place at the molecular or supramolecular level [24,25]. It can occur by self-association or by an association with other structures through interactions such as hydrogen bond, van der Waals forces, and ionic or hydrophobic interactions. It can also be caused by an inclusion/complexation mechanism, like the iodine inclusion complex with starch [25].

CS self-assembled (also referred to as self-aggregated) nanoparticles (NPs) are particularly useful for encapsulating hydrophilic as well as lipophilic drugs [26]. Self-assembly can be provoked by the introduction of hydrophobic moieties into the CS molecules by grafting, in order to modify its hydrophobic-hydrophilic balance. The grafting agent can be a hydrophobic moiety, such as cholesterol [27], cholic [28], and deoxycholic acid [29], or 5β-cholanic acid [30], to form micelles that can entrap lipophilic drugs or it can be the drug itself. Frequently, a water soluble CS derivative, such as glycol chitosan [31] or succinyl chitosan [32], is used instead of CS. Another suitable way to generate self-assembled chitosan nanoparticles is through the formation of polyelectrolyte complexes with polyanions [33]. The aim of the present article is to review the main approaches used for preparing chitosan nanoparticles by self-assembly through both procedures, and to illustrate the state of the art in drug delivery.

## 2. Polyelectrolyte Complexes

Polyelectrolyte complexes (PECs) are formed when the solutions of two polyelectrolytes carrying complementary charges (i.e., a polycation and a polyanion or their corresponding salts) are mixed together. PEC formation is mainly caused by the strong Coulomb interaction between the oppositely charged polyelectrolytes. The formation of complexes brings about at least a partial charge neutralization of polymers [10]. The complexes obtained (also called polysalts) generally precipitate or separate from the solution forming a complex rich liquid phase (coacervate). However, under certain conditions, the polyelectrolytes, with weak ionic groups and significantly different molecular weights at non-stoichiometric mixing ratios, can generate water-soluble PECs on a molecular level [34,35].

**Figure 2:**
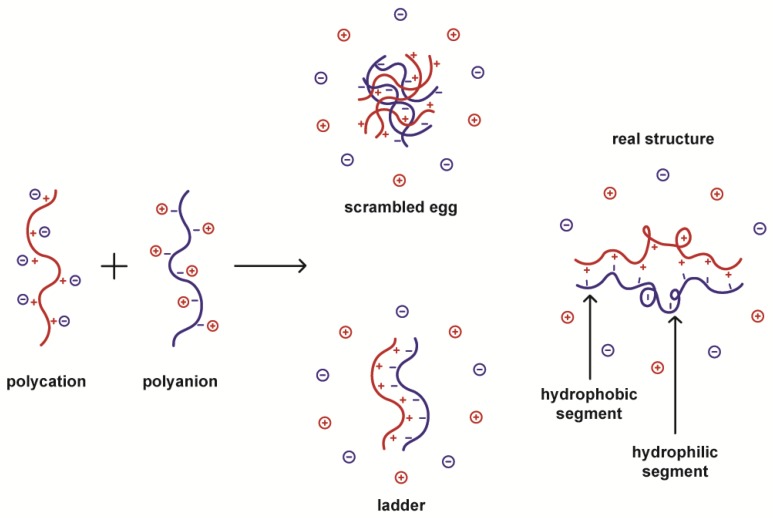
The structure of polyelectrolyte complexes. Scrambled egg and ladder arrangements illustrate extreme situations. The actual structure can be represented as an intermediate one combining hydrophobic ladder-like segments coexisting with disordered hydrophilic regions.

The formation of polyelectrolyte complexes is accompanied by the release of small counter-ions into the medium. The increase in entropy produced by the release of these low molecular weight counter-ions to the medium is the main driving force for PEC formation. Although the electrostatic interaction between the complementary ionic groups of polyelectrolytes is responsible for PEC formation, hydrogen bonds, and hydrophobic interactions also contribute to complexing. The arrangement of chains in a PEC can be envisaged as a combination of a disordered scrambled egg-like structure and a highly ordered ladder-like organization (Figure 2). Therefore, the actual structure having hydrophobic and hydrophilic regions makes PECs a particular class of physically cross-linked hydrogels that are sensitive to pH and to other environmental factors such as temperature and ionic strength.

Many factors affect the structure and stability of PECs; these include: the degree of ionization of each one of the polyelectrolytes and their charge density and charge distribution on the polymer chains, polyelectrolytes concentration, mixing ratio (*Z*), mixing order, the nature of the ionic groups on the polymer chains, molecular weights of the polyelectrolytes, flexibility of the polymer chains, interaction time and temperature and ionic strength, as well as the pH of the medium [36].

As a cationic biopolymer, CS may react with negatively charged polyelectrolytes, giving rise to the formation of PECs [37,38]. Many reports show PECs produced with chitosan and carboxymethyl cellulose (CMC) [39,40], alginate [41,42,43,44,45], poly(acrylic acid) [46,47], pectin [48,49,50,51], carrageenans [52,53], heparin [54], and other polyions [55,56,57,58,59,60,61].

### 2.1. Chitosan Based PEC Nanoparticles and Their Application in Drug Delivery

Because of the above mentioned biological properties of CS, many applications of these PECs have been proposed for biomedical purposes, particularly for drug delivery [62]. Hence, researchers have shown special interest in the preparation of chitosan PEC nanoparticles for the delivery of drugs, proteins, genes, and vaccines [36,63,64].

**Figure 3:**
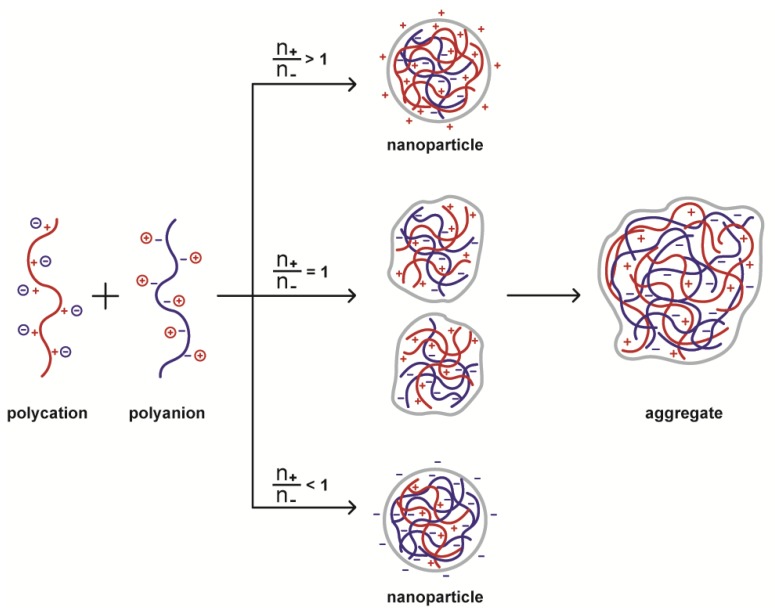
Effect of the polyelectrolytes charge ratio on the size and charge of the polyelectrolyte complexes (PEC) formed. When the charge ratio is different from one, the nanoparticles formed are charged with the same charge as the polyion in excess. If the charge ratio equals one, uncharged particles are formed, thereby producing large aggregates.

When chitosan PEC particles are formed, they tend to aggregate because of charge neutralization, therefore, at least two conditions are mandatory in order to avoid aggregation and to obtain nanoparticles: the polyelectrolyte solutions must be diluted, and one of the polyions must be in the appropriate excess required so that the charge ratio (*n*_+_/*n*_−_) ≠ 1 (Figure 3).

Other conditions, such as pH (particularly important in weak polyelectrolyes), ionic strength, and the mixing rate, should be adjusted to the particular chitosan-polyanion pair system selected, since these variables will also influence the size and charge of nanoparticles.

Different preparation methods will result in diverse kinds of nanoparticles, which can be classified as nanoaggregates, nanocapsules or nanospheres. The particular procedure selected can be largely determined by the water solubility of the active agent that will be encapsulated and the polyanion used.

#### 2.1.1. Chitosan-Alginate PEC Nanoparticles

**Figure 4:**
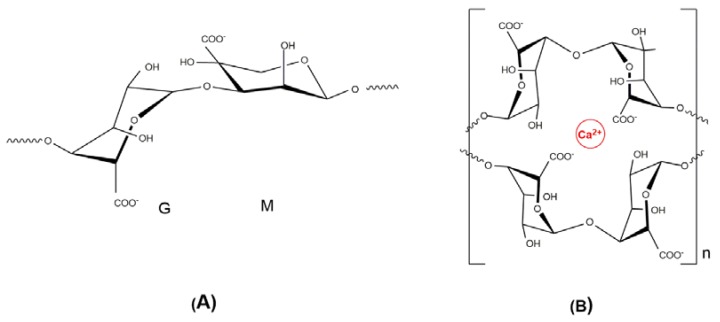
(**A**) Structural units in alginate. (G) Guluronic acid; (M) Mannuronic acid; (**B**) Representation of two G-blocks forming an ‘egg box’ sequence with a calcium ion.

Alginates are a family of anionic polysaccharides extracted from brown algae. They are composed of α-l-guluronic acid (G) and β-d-mannuronic (M) acid units that are linearly linked by 1,4-glycosidic bonds (Figure 4). The M/G ratio and their distribution along the chains (chain microstructure) are strongly dependent on the particular species of algae from which it was extracted [65]. Alginate (ALG) is non-toxic, biocompatible and biodegradable, mucoadhesive, and non-immunogenic. The gelling capacity of ALG in the presence of calcium ions in the so-called “egg-box” model has been extensively employed to prepare gels, capsules, and micro- and nanoparticles for drug delivery [66]. The guluronic units are responsible for the crosslinking reaction; and, the properties of the beads formed, such as strength and porosity, will therefore depend on the alginate source. Other parameters affecting the characteristics of beads are ALG molecular weight, and the concentration of CaCl_2_ and alginate solutions [65].

Chitosan-alginate PEC nanoparticles are usually prepared by one of the following three procedures.

##### Plain Complex Coacervation by Mixing Dilute Solutions of CS and ALG

In this procedure, the order of addition of one polysaccharide into the other, the CS/ALG ratio, the molecular weight of both polyelectrolytes and the pH and the ionic strength of the solutions are important factors in determining the relevant parameters of nanoparticles (size, particle charge, stability, encapsulation efficiency).

This procedure was used by Liu and Zhao [67] to prepare negatively or positively charged CS/ALG nanoparticles by dropping a CS solution over the ALG solution. They found that particle sizes varied from 320 to 700 nm, depending on the pH and the ionic strength of the solution. At pH 4.80 in deionized water the sample displayed a narrow unimodal size distribution with an average hydrodynamic diameter (Dh) of 329 ± 9 nm. The Zeta-potential of NPs was also dependent on pH and ranged from +6.34 mV at pH 3.0 to –44.5 mV at pH 10.0. The loading capacities of NPs for ibuprofen and dipyridamole were 14.18% and 13.03%, respectively. Drug release was governed simultaneously by the solubility of the drug and the permeability of the CS/ALG nanoparticles [67].

In a modification of this procedure, a CS solution containing Tween 80 (stirring the chitosan solution with Tween 80 generated chitosan drops) was dropped into a previously prepared solution of an alginate complex with doxorubicin (DOX). The NP suspension was stirred overnight and the doxorubicin loaded CS/ALG NPs were separated by centrifugation. The size of the NPs was 100 ± 35 nm, with a polydispersity index (PDI) of 0.40 ± 0.07, the Zeta-potential was of +35 ± 4 mV, and the encapsulation efficiency (EE) achieved was 95 ± 4% for the optimal formulation (CS/ALG = 2:1) [68].

The reverse procedure was used to encapsulate amoxicillin in CS/ALG nanoparticles. Essentially, a mixture of chitosan, Pluronic F-127 (surfactant), and amoxicillin was prepared in various concentrations of all the components. An aqueous solute on of ALG was sprayed into this mixture with stirring to form NPs. Both of the solutions were at pH 5.0. The process was optimized for variables such as pH and the mixing ratio of polymers, concentrations of polymers, drug and surfactant, using the 33 Box-Behnken design. The resulting particle size, surface charge, drug entrapment percentage, in vitro mucoadhesion, and in vivo mucopenetration of nanoparticles in rat models were analyzed. The optimized formulation with particle size, Zeta-potential and encapsulation efficiencies of 651 nm, +59.76 mV and 91.23%, respectively, showed comparatively low in vitro mucoadhesion as compared to plain chitosan nanoparticles, but excellent mucopenetration and localization [69].

A modified hybrid blending system was developed by Goycoolea et al., which combined the complex coacervation of CS and ALG with the ionotropic gelation of CS with trisodium tripoliphosphate (TTP). The purpose of this combination was to increase the stability in the biological media and for better pharmacological performance than with conventional CS-TPP nanoparticles. In this method, an ALG solution containing TPP was mixed under rapid stirring with the CS solution forming the CS-TPP-ALG nanoparticles. Insulin loaded CS-TPP-ALG nanoparticles were obtained by adding insulin into the ALG-TPP solution before mixing with the CS solution. The average particle size of the insulin-loaded CS-TPP-ALG NPs was 297 ± 4 nm (PDI 0.25), similar to that of the unloaded NPs, which was 307 ± 5 nm (PDI 0.22). High positive Zeta-potential values ~ +42 mV were obtained in both cases, providing good stability to the NPs. Insulin loading efficiencies (defined as insulin loaded per weight of nanoparticles) as high as 50.7% were attained [70].

##### Ionotropic Pregelation of Alginate Followed by Complexation with Chitosan

This is a very common method in which pregelation is usually attained with CaCl_2_, but other divalent ions may also be used. The active agent can be dissolved or dispersed in the ALG solution or it can be loaded into the resulting CS/ALG nanoparticles. Azevedo et al. used this procedure by setting the initial pH of the ALG and CS solutions to 4.9 and 4.6, respectively. In their formulation, the average size of CS/ALG NPs was 120 ± 50 nm with a Zeta-potential of −30.9 ± 0.5 mV. Vitamin B2 loaded NPs were obtained by dissolving the compound in the ALG solution before the pregelation step. The average size of nanoparticles with vitamin B2 was 104 ± 67 nm (PDI 0.32 ± 0.07) with a Zeta-potential of −29.6 ± 0.1 mV. The nanoparticles showed EE and loading capacity (LC) values of 56 ± 6% and 2.2 ± 0.6%, respectively [71].

##### Oil-in-Water (*O/W*) Microemulsion of Alginate Followed by Ionotropic Gelation and Further Complexation with Chitosan

This method is usually preferred for encapsulating hydrophobic drugs. The preparation of nanocapsules is carried out by emulsifying a solution of the drug (oil phase) into the aqueous sodium alginate solution containing a surfactant, followed by gelation with calcium chloride and CS.

Bhunchu et al. used this method to prepare CS/ALG NPs containing curcumin diethyl disuccinate (CDD). CDD dissolved in acetone (1 mL) was dropped into 20 mL of a dilute ALG solution (0.6 mg/mL) containing a non-ionic surfactant (Pluronic F127, Cremophor RH40™ and Tween 80^®^). Four mL of a CaCl_2_ solution (0.67 mg/mL) was added while stirring, followed by sonication. Four mL of the CS solution at various concentrations (0.15–0.45 mg/mL in 1% (*v/v*) acetic acid) were added with continuous stirring at 1000 rpm for 30 min. After standing overnight for equilibration the CDD loaded CS/ALG NPs were obtained as dispersion in the aqueous solution. Pluronic F127 gave the smallest particle size, 414 ± 16 nm (PDI 0.63 ± 0.05) with the highest Zeta-potential, −22 ± 1 mV. The EE and LC of these NPs were 55 ± 1% and 3.33 ± 0.08%, respectively. These NPs improved cellular uptake of CDD in Caco-2 cells, as compared to free CDD [72].

A list of some selected examples of CS/ALG PEC nanoparticles based on the different procedures mentioned above is given in Table 1.

Inspection of Table 1 reveals that a wide variation in particle size and Zeta-potential is reported for all of the three general procedures devised for preparing CS-ALG PEC nanoparticles. The same happens with the EE and the LC. This is a consequence of the already mentioned dependence of these parameters on multiple variables.

In plain complex coacervation a surfactant is sometimes added to improve the entrapment efficiency and the solubility of the drug [69,73], but it might increase the size of the particles and decrease the Zeta-potential [69]. EE values that are reported in these methods vary from around 50% [69,70] to 95% [67]. The LC is not always declared, but the values of 14.18% and 13.03%, depending on the drug [66], have been reported.

In the method based on the pregelation of alginate encapsulation efficiencies reported are in general higher than 50%. For instance, Azevedo et al. [71] reported an EE of 56 ± 6% for vitamin B2, while other authors declared 73 ± 2% for insulin [75], 62% for acetamiprid [76], and 95.6% for EGF-antisense [77]. However, loading capacities reported were only 2.2 ± 0.6% [71] and 10 ± 2% [75]. For the NPs that are loaded with vitamin B2 the PDI was 0.32 ± 0.07. The other reports did not declare the PDI obtained.

The method based *o/w* microemulsion of ALG followed by ionotropic gelation and complexation with CS produced in general nanocapsules with sizes of about 400 to 660 nm [72,79,80]. The PDI was reported only in reference [74] and was 0.63 ± 0.05. When using low molecular weight polysaccharides, particles sizes ranging from 134 to 229 nm were reported [81]. Encapsulation efficiencies informed were 55 ± 1% for curcumin diethyl disuccinate (LC, 3.33 ± 0.08%) [72] and 88.4% for BSA [81].

#### 2.1.2. Chitosan-Pectin PEC Nanoparticles

**Figure 5:**
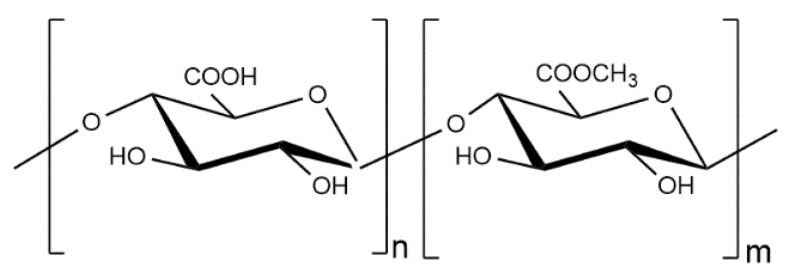
Chemical structure of partially acetylated polygalacturonic acid in pectin.

Pectin is an anionic hetero-polysaccharide derived from plant cell walls, consisting primarily of 1,4 linked α-d-galactopyranosyl uronic acid residues with 1,2-linked α-l-rhamnopyranose residues interspersed with varying frequencies (Figure 5). Pectin structure also presents a certain amount of neutral sugars (arabinose, galactose, rhamnose, xylose, and glucose). A number of galacturonic acid residues in the pectin are methyl or acetyl esterified. The percentage of galacturonic acid residues that are esterified is known as the degree of esterification (DE).

Pectin is hydrophilic, biocompatible, and biodegradable, and it has low toxicity. As in ALG, pectin with a low methoxyl content (DE < 50%), has the ability to gel in the presence of Ca^2+^ ions generating junction zones between chains with an egg-box structure. Pectins with higher DE can also form gels, provided that there are a sufficient number of blocks of non-esterified uronic acid residues per molecule to allow the formation of a sufficient number of junction zones to form a network. These properties of pectin have been employed to prepare diverse formulations for drug delivery applications.

Galacturonic acid provides pectin with a negative charge in solutions with pH higher than 3.5, permitting the formation of polyelectrolyte complexes with chitosan. The strength of the interaction depended on the degree of esterification of the pectin, with pectins of a relatively low DE (36%) readily forming PECs with CS [82]. PEC formation is also affected by the ratio of pectin to CS and the pH of the solutions [83].

CS-pectin PEC nanoparticles can be prepared by the same methods previously described for CS-ALG PEC nanoparticles. Birch and Schiffman prepared nanoparticles by the complex coacervation technique adding pectin at the appropriate CS-to-pectin ratio to the CS solution. They thereby obtained particle sizes ranging from 560 ± 10 nm to 1000 ± 40 nm. The Zeta-potential varied from +20 ± 1 mV to +26 ± 1 mV. When the addition order was reversed the particle size increased from 460 ± 20 nm to 1110 ± 30 nm and the Zeta-potential changed from +19 ± 1 to +28 ± 1 mV [84].

Rampino et al. prepared CS-pectin PEC nanoparticles by two different procedures: a) coating, by adding a dispersion of low molecular weight CS NPs previously prepared by the ionotropic gelation of CS with TPP to a pectin (from apple and citrus fruit) solution; and, b) blending, by adding a CS solution to a solution of pectin and TPP. Nanoparticles were charged with ovalbumin (OVA) and bovine serum albumin (BSA) as the model proteins. They pointed out that the blending technique could be advantageous because, by being a one-step preparation, it is highly desirable for a scale-up process. Additionally, it gives the possibility of tuning the size and Zeta-potential by properly selecting the ratios of CS, pectin, and TPP. However, they found that there was a decrease in the loading of BSA and OVA in the case of the blending technique (loading efficiency, ranging between 16% and 27%) due to the electrostatic interactions of CS with the protein and pectin, both negatively charged. Therefore, they concluded that the selected technique would depend on the physicochemical characteristics of the polymer and the protein involved [85]. Some of the parameters reported in their work are listed in Table 2, together with some selected examples of CS-pectin preparation procedures reported by other authors.

Not all of the references in Table 2 report parameters, such as EE, LC, and PDI. In plain complex coacervation, Maciel, et al. [23] reported microparticles with size less than ~2500 nm using charge ratios (*n*_+_/*n*_−_ given by the chitosan/pectin mass ratio) of 0.25 and 5.00, with PDIs of 0.25 ± 0.06 and 0.40 ± 0.06, respectively. The highest EE (~62.0%) of the system was observed at a charge ratio (*n*_+_/*n*_−_) 5.00. Andriani et al. [87] added glutaraldehyde to the chitosan-pectin mixed solution. This way, they obtained encapsulation efficiencies varying from 24.0% (LC 6.30%) to 94.7% (LC 21.05%). Combining ionotropic gelation and complex coacervation, Al-Azi, et al. [86] reported a PDI of 0.67–0.71. Insulin association efficiency varied from 2.40 ± 0.33% (LC 0.31 ± 0.04%) at pH 3, to 4.06 ± 0.12% (LC 0.52 ± 0.01%) at pH 5. Using Ca^2+^ ions caused a marked improvement in insulin association efficiency of nanoparticles.

#### 2.1.3. Chitosan-Dextran Sulfate PEC Nanoparticles

**Figure 6:**
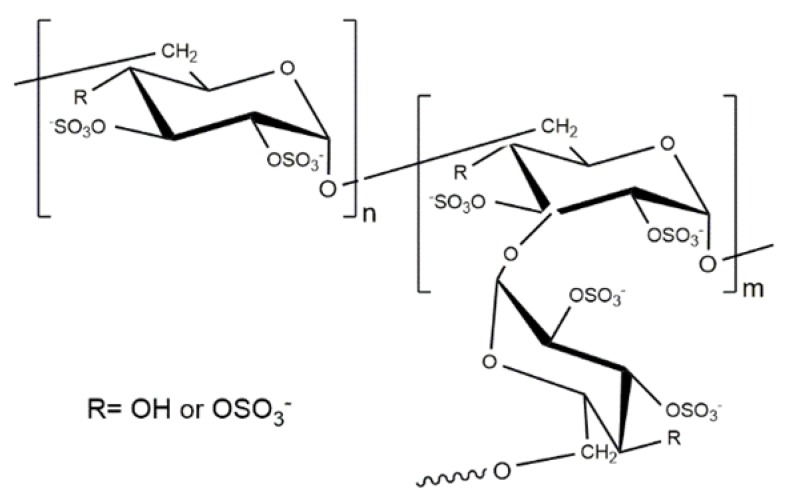
The chemical structure of dextran sulfate.

Dextran sulfate (DS) is a biodegradable and biocompatible negatively charged branched polyanion that is able to interact with positively charged polymers. It is a high-molecular weight, branched-chain polysaccharide polymer of d-glucose containing 17–20% sulfur. The straight chain consists of approximately 95% α-(1,6) glycosidic linkages. The remaining α-(1,3) linkages account for the branching of dextran (Figure 6).

DS has been used as an anticoagulant and with applications in drug delivery. For instance, it was used to mask the positive charge of doxorubicin (DOX) before its addition to a CS solution for nanoparticle formation by ionotropic gelation with TPP. This modification doubled DOX EE relative to the controls, and made it possible to reach loadings of up to 4.0 wt % DOX [89].

CS-DS PEC nanoparticles are almost invariably prepared by simple coacervation. The factors affecting the mechanism for the formation of these nanoparticles: the mode of addition, charge mixing ratio, pH and ionic strength of the media, and the molar mass of both components have been thoroughly reviewed by Schatz et al. [90,91].

There are numerous reports on the preparation of CS-DS PEC nanoparticles with a potential application for the delivery of proteins (insulin, BSA), growth factors [92,93,94], immunoglobulin-A [95], and vaccines [96,97]. Recently, fluorescein isothiocyanate loaded CS-DS nanoparticles (FCS-DS NPs; mean size, 400 nm (PDI 0.25 ± 0.01); and, surface charge, +48 mV) were topically applied to the porcine ocular surface where it was retained for more than 4 h. After 6 h under the topical FCS-DS NPs treatment, particles were accumulated in the corneal epithelium but were not found in the corneal stroma. However, when the epithelium was removed, the FCS-DS NPs penetrated the stroma. These results indicate that FCS-DS NPs are potentially useful for drug/gene delivery to the ocular surface and to the stroma when the epithelium is damaged [98].

Most of nanoparticles formulations reported describe processing factors affecting the characteristics of CS-DS nanoparticles, including their physicochemical properties as well as the optimal conditions for their preparation. Some examples are listed in Table 3.

In Table 3, reference [99] illustrates that different results are obtained for the same system when used to encapsulate two different substances, BSA and Rhodamine 6G, by complex coacervation. In this work, the size of the BSA loaded CS-DS NPs varied from 478 nm (PDI 0.64; EE 96.8%; LC 81.6%) to 1138 nm (PDI 0.97; EE 53.2%; LC 29.3%). However, the Rhodamine 6G loaded nanoparticle sizes were higher, varying from 545 nm (PDI 0.60; EE 98%; LC 31%) to 3521 nm (PDI 0.68; EE 42%; LC 18%). In both cases, the bigger NPs were more polydisperse and had lower LC and EE [99]. 

Sarmento et al. prepared CS-DS PEC nanoparticles containing insulin with association efficiencies varying from 85.4 ± 0.5% to 72 ± 3%, depending on CS/DS mass ratio [100]. In a later article [101], these authors evaluated the pharmacological activity of insulin-loaded CS-DS PEC nanoparticles following oral dosage in diabetic rats. On this occasion, they introduced small changes in the preparation parameters and obtained somewhat lower association efficiencies, ranging from 69 ± 1% (LC, 2.3 ± 0.6%) to 24 ± 2% (LC, 2.0%) [101]. This influence of the preparation parameters on the characteristics of the PEC nanoparticles can be used to modify them to meet the specific requirements of a determined application.

PECs of soluble chitosan derivatives with DS have also been formulated to overcome the insolubility of chitosan in neutral and basic media. Glycol chitosan (GC) and DS solutions were mixed together to prepare GC-DS PEC nanoparticles that were loaded with the antifolic agent methotrexate (MTX), aiming to increase its efficacy for the treatment of brain tumors. EE was as high as 87% for a particle size of 149 ± 41 nm (PDI 0.7 ± 0.1). In vitro experiments indicated their potential for the controlled delivery of the drug to the brain [103]. 

PEC nanoparticles of water soluble *N*,*N*,*N*-Trimethyl chitosan (TMC) and DS were prepared by adding DS solutions to TMC solutions at the desired pH values (5, 8, 10, and 12). The release efficiency and ex vivo nasal toxicity evaluation were assessed after loading a model drug, ropinirole hydrochloride, into an optimized PEC formulation at pH 10 (particle size, 255 ± 10 nm; Zeta-potential, −4 ± 1 mV; LC = 82 ± 2%; EE = 87.9 ± 0.6%). Data indicated that the PECs produced at alkaline pH have a reliable formulation for nasal administration. They are biologically compatible with the mucosal surface, thereby being potentially applicable as carriers for nose to brain drug delivery [104].

#### 2.1.4. Chitosan-Carboxymethyl Chitosan PEC Nanoparticles

**Figure 7:**
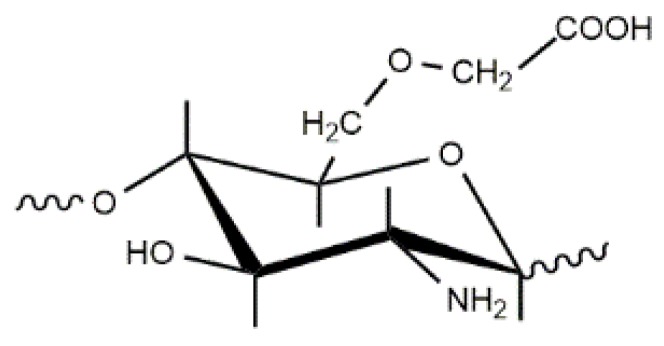
The structural unit of carboxymethyl chitosan.

*O*-Carboxymethyl chitosan (CMCS) is a water-soluble amphiphilic derivative of chitosan that conserves the biological properties of native chitosan with increased antibacterial activity [105]. The structural unit of CMCS is shown in Figure 7. CMCS has been applied in biomedicine, especially in drug delivery where CMCS nanoparticles prepared by ionotropic gelation have demonstrated promising results for drug [106,107] and antigen delivery [108].

The p*K*a of CMCS is 2.0–4.0, so that at pH above 4 it is negatively charged and forms polyelectrolytes complexes with chitosan [109]. CS-CMCS PEC nanoparticles were produced by complex coacervation. Wang et al. developed insulin-loaded nanogels with opposite Zeta-potential by adding a previously prepared insulin-CMCS solution into a CS solution (particle size, 260 ± 5 nm; PDI 0.08 ± 0.02; Zeta-potential, +17.2 ± 0.5 mV for insulin: CMCS/CS-NGs(+)) or inversing the order of addition (particle size, 243 ± 4 nm; PDI 0.03 ± 0.01; Zeta-potential, −15.9 ± 0.5 mV for insulin: CMCS/CS-NGs(−)), respectively. Encapsulation efficiencies of about 75% and loading capacities near 30% were attained in both cases. They observed that negatively charged particles exhibited enhanced mucoadhesion in the small intestine and had better intestinal permeability in the jejunum, indicating there was a better performance in insulin: CMCS/CS-NGs(−) for blood glucose management than in those positively charged [110,111].

CS-CMCS nanoparticles have also been prepared by combining ionotropic gelation and complex coacervation. CMCS and TPP at varying concentrations were blended with a previously prepared mixture of DOX and CS solutions. Nanoparticles sizes between 249 ± 10 nm (Zeta potential, −27.6 ± 0.8 mV) and 362.7 ± 8.4 nm (Zeta potential, −42 ± 1 mV) with encapsulation efficiencies and loading capacities of around 70.5% and 20%, respectively, were obtained depending on the preparation conditions. Results from in vivo experiments indicated that CS/CMCS-NPs were efficient and safe for the oral delivery of DOX [112]. After certain modifications of the preparation procedure, positively charged CS/CMCS-NPs were obtained. This time, the DOX aqueous solution was premixed with CMCS and the CS solution and TPP were subsequently blended with the mixture under agitation. Nanoparticles sizes were of between 197 ± 11 nm (PDI 0.235; Zeta-potential, +37.6 ± 0.8 mV) and 442 ± 7 nm (PDI 0.635; Zeta-potential, +12.2 ± 0.6 mV), depending on the pH of the medium. In vivo studies revealed that CS/CMCS-NGs had a high transport capacity by paracellular and transcellular pathways, which guaranteed the excellent absorption of encapsulated DOX throughout the entire small intestine [113].

#### 2.1.5. Chitosan-Chondroitin Sulfate PEC Nanoparticles

**Figure 8:**
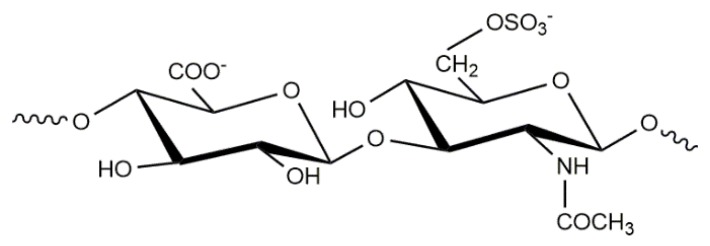
Chemical structure of chondroitin sulfate.

Chitosan-chondroitin sulfate PEC NPs have been prepared by complex coacervation and the influence of the preparation conditions on the properties of nanoparticles was reported [114,115]. Chondroitin sulphate is a linear glycosaminoglycan (GAG) that is composed of alternating d-glucuronate and β(1,3) linked *N*-acetyl-d-galactosamine-4- or 6-sulfate (Figure 8). It is found in cartilage, bone and connective mammalian tissue. Chondroitin sulphate (CHOS) has shown in vivo anti-inflammatory properties in animal models and in vitro regulation of chondrocyte metabolism, such as the stimulation of proteoglycan and collagen synthesis and the inhibition of the production of cytokines that are involved in cartilage degradation [116]. Its biological properties have stimulated the preparation and evaluation of CS-CHOS nanoparticles for drug/gene delivery [117,118] and delivery of platelet lysates [119]. CS-CHOS nanoparticles have been suggested as a novel delivery system for the transport of hydrophilic macromolecules [120].

#### 2.1.6. Chitosan-Heparin and Chitosan-Hyaluronan PEC Nanoparticles

**Figure 9:**
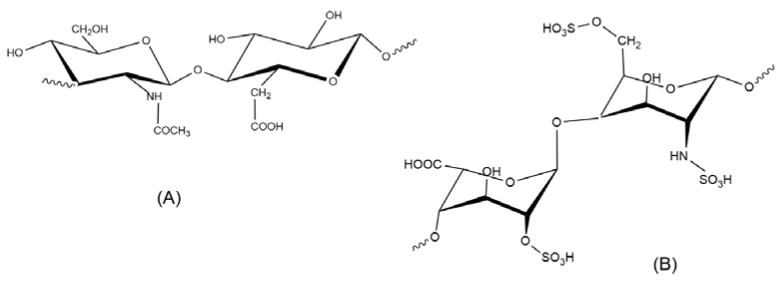
Chemical structures of (**A**) Hyaluronic acid and (**B**) Heparin.

CS PECs with other two glycosaminoglycans, hyaluronic acid (hyaluronan, HA) and heparin (HEP), have also been used to prepare nanoparticles. HA is a high molecular weight linear polysaccharide that is composed of β(1,3) linked d-glucuronate and *N*-acetyl-d-glucosamine units. It is present in all soft tissues of higher organisms, and in particularly high concentrations in the synovial fluid and vitreous humor of the eye. It plays a vital role in many biological processes, such as tissue hydration, proteoglycan organization, cell differentiation, and angiogenesis, and acts as a protective coating around the cell membrane. On the other hand, HEP has a more heterogeneous composition, but its main disaccharide unit is composed of d-glucuronate-2-sulfate (or iduronate-2-sulfate) and α(1,3) linked *N*-sulfo-d-glucosamine-6-sulfate, which provides it with the highest negative charge density of any known biological macromolecule (Figure 9). HEP can be primarily found on the cell surface or in the extracellular matrix, attached to a protein core. Heparin is a well-known anticoagulant drug and is extensively used in medical practice [121]. The important bioactivity of both GAGs has stimulated the preparation of CS-HA and CS-HEP PEC nanoparticles due to their high potential in applications as delivery systems for these macromolecules, particularly in tissue engineering [58,122,123,124].

#### 2.1.7. Chitosan and Poly(γ-Glutamic Acid) PEC Nanoparticles

**Figure 10:**
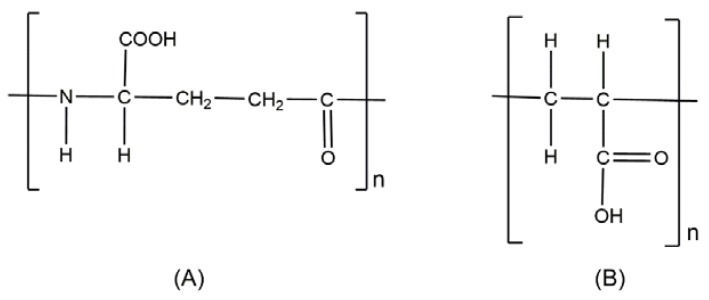
Chemical structures of (**A**) Poly(γ-glutamic acid) and (**B**) Poly(acrylic acid).

Poly(γ-glutamic acid) (γ-PGA) is an anionic, natural polypeptide that is made of d- and l-glutamic acid units, joined together by amide linkages between the α-amino and γ-carboxylic acid groups (Figure 10). PEC formation between CS and γ-PGA has been evaluated in terms of physical and chemical properties. In experimental trials, it has shown wound-healing efficacy with a potential application as a wound dressing material [125].

PEC nanoparticles of γ–PGA and low molecular weight CS were obtained by complex coacervation by Lin et al. by adding an aqueous γ-PGA solution at pH 7.4 to a low molecular weight CS solution at different pH values. The NPs prepared at pH 6.0 and a CS/γ-PGA ratio of 4.5:1.0 (*w/w*) had a Zeta-potential of +32 ± 2 mV with a particle size of 146 ± 2 nm (PDI 0.21 ± 0.02). Insulin loaded NPs were obtained by including insulin in the γ–PGA solution before its addition to the CS solution. Nanoparticles with a mean size of 198 ± 6 nm (PDI 0.30 ± 0.09) and a Zeta-potential of 28 ± 1 mV were obtained when the amount of insulin added was 84 μg/mL (EE 55 ± 3, LC 14.1 ± 0.9. Animal studies indicated that the insulin loaded NPs enhanced insulin adsorption and reduced the blood glucose level in diabetic rats [126]. Hajdu et al. [127] reported the effect of pH, polymer ratios, concentrations, and orders of addition on the physicochemical properties of NPs.

The same procedure was used to prepare exendin-4 loaded NPs, only that in this case, the CS solution contained distinct metal ions (Cu^2+^, Fe^2+^, Zn^2+^ or Fe^3+^) to enhance the drug loading efficiency. Loading efficiency of 61 ± 2% (LC 15 ± 2%) was achieved for exendin-4 loaded NPs formed with Fe^3+^. Their particle size was 261 ± 26 nm [128].

Nanoparticles of γ-PGA and CS have also been prepared by the combination of ionotropic gelation and complex coacervation. To this end, the insulin and γ-PGA solutions were premixed. Afterwards, TPP and MgSO_4_ solutions were mixed together and were added to the insulin and γ-PGA mixture. The resulting solution was then added by flush mixing with a pipette tip into the aqueous CS solution and the nanoparticles were then formed. These NPs also resulted in a promising carrier for the improved trans mucosal delivery of insulin in the small intestine [129,130].

More recently Pereira et al. used the pregelation method to prepare CS/γ-PGA PEC nanoparticles to be used as a nanocarrier system for the plant growth regulator gibberellic acid (GA3). To this end, a CaCl_2_ solution was added to a solution of γ-PGA at pH 4.9. Then, a CS solution at pH 4.5 was added to the γ-PGA/CaCl_2_ solution while stirring, using a peristaltic pump. To prepare GA3 loaded NPs, the plant hormone was added to the γ-PGA/CaCl_2_ before the addition of the CS solution. The unloaded γ-PGA/CS nanoparticles presented an average size of 117 ± 9 nm (PDI 0.43 ± 0.07) and Zeta-potential of −29.0 ± 0.5 mV at pH 4.4. The corresponding values for the GA3 loaded γ-PGA/CS nanoparticles were 134 ± 9 nm (PDI 0.35 ± 0.05) and −27.8 ± 0.5 mV at pH 4.4, respectively. The encapsulation efficiency of the GA3 particles was 61%. In laboratory experiments using *Phaseolus vulgaris* seeds, the γ-PGA/CS-GA3 NPs showed high biological activity, with an enhanced rate of germination when compared with the free hormone. The encapsulated GA3 was also more efficient than the free GA3 in the increase of leaf area and the induction of root development, demonstrating the considerable potential of this system for its use in the field [131].

#### 2.1.8. Chitosan-Poly(Acrylic Acid) PEC Nanoparticles

Poly(acrylic acid) (PAA) is a biocompatible linear anionic polyelectrolyte that readily reacts with CS, generating polyelectrolyte complexes by the electrostatic interaction between its COO^− ^ groups and the NH_3_^+^ groups of chitosan [33,38].

Hu et al. prepared CS-PAA PEC nanoparticles by template polymerization of acrylic acid in chitosan solution using chitosan as the template. Positively charged NPs with the mean size and Zeta-potential of 206 ± 22 nm and +25.3 ± 3.2 mV, respectively, were obtained with 70% yield. These NPs were loaded with silk peptide powder (SP) with an encapsulation efficiency of 82%. Release experiments showed a marked pH dependence of the peptide release profile. They also obtained CS-PAA PEC NPs by complex coacervation by dropping the CS solution into the solution of PAA and vice versa, to study the effect of reversing the order of addition on the resulting nanoparticles. When CS was added to PAA, negatively charged particles were obtained with a mean size of 436 ± 78 nm and a Zeta-potential of −22.2 ± 3.6 mV. On the other hand, adding PAA solution into the CS solution produced positively charged NPs with a mean size and Zeta-potential of 358 ± 46 nm and +47 ± 3 mV, respectively. The order of addition also influenced the microstructure of NPs. Transmission electron micrographs of dry nanoparticles showed that NPs that were obtained by adding the CS solution over the solution of PAA had a hollow core, in contrast with the nanoparticles obtained with the reverse addition method, which presented a compact core [132]. In a further study, it was found that nanoparticle size was affected by the molecular weight of CS and PAA, the ratio of the amino group to the carboxyl group (*n*_a_/*n*_c_) and incubation temperature [133].

Davidenko et al. examined the influence of some experimental parameters such as the pH of the polyelectrolyte solutions, their concentrations and the purification procedure on the dimensions of nanoparticles and their size distribution. NPs were formed by the dropwise addition of an aqueous solution of PAA into the corresponding volume of an aqueous solution of CS of a determined concentration with high-speed magnetic stirring (ca. 1300 rpm). The ratio of primary amino groups in CS to carboxylic groups in PAA was fixed at 1.25. They showed that it was possible to obtain nanometric particle suspensions at concentrations of below 0.1%. The most convenient pH values for obtaining CHI-PAA NPs with an optimum yield (nearly 90%) are 4.5–5.5 for CS and 3.2 for PAA. Under these conditions, the size of NPs was 0.477 ± 0.008 nm. Particle sizes of approximately 130–140 nm were obtained at other pH values, but yields were lower than 45%. It was found that purification by dialysis could provoke a drastic change both in the distribution profile and in the particle size of the complex. To avoid this the pH of the NPs dispersion should be as near as possible to the pH of the outer dialysis solution [134]. CS-PAA PEC nanoparticles obtained by this procedure were loaded with 5-fluoruracil (5-Fu) and the release profiles at pH 2 and 7.4 were obtained. At pH 2 almost 100% release was achieved after two hours, whereas at pH 7.4 only 65% of the loaded drug was released after nine hours. At this pH constant release was observed after the first 90 min [135].

The complex coacervation procedure has also been used for preparing CS-PAA PECs nanofiber structures with average fibre diameters of 210 to 910 nm and Zeta-potentials of +39 ± 1 mV to −22 ± 3 mV, respectively. These parameters vary according tothe preparation conditions (volume ratio of CS to PAA, final suspension pH, concentration and molecular weight of CS, incubation time and reaction temperature). Nanofibers can bind plasmid DNA very well and show a potential to enhance gene transfer in tissue engineering applications [136,137].

#### 2.1.9. Chitosan PEC Nanoparticles with Other Polyanions

The preparation of CS PEC nanoparticles for the delivery of drug and therapeutic proteins is continuously increasing. They include other polyanions of natural origin, like carrageenan [138,139], carboxymethyl gum kondagogu [140], and gum arabic [141], as well as synthetic ones. Examples of the latter are poly(malic acid) [142], poly(2-acrylamido-2-methylpropanesulfonic acid) [143], and polystyrene-block-poly(acrylic acid) [144]. The methods that were used for the preparation of these nanoparticles are based on the general techniques already described, and will therefore not be discussed here.

## 3. Hydrophobic Modification of Chitosan and Derivatives for Self-Assembly

**Figure 11:**
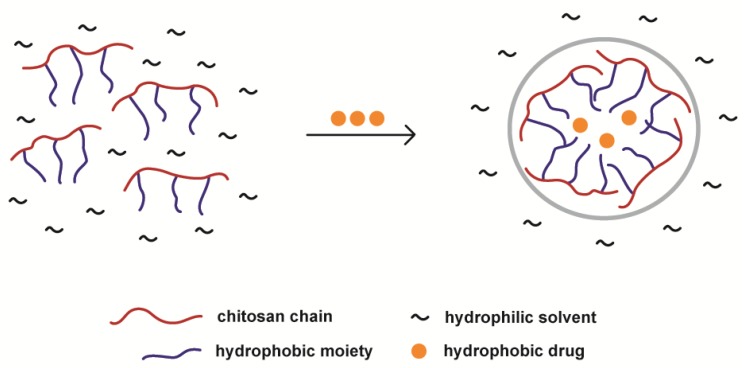
Schematic representation of hydrophobically modified chitosan self-assembly. Aggregates can entrap hydrophobic drugs in their hydrophobic core.

The hydrophobic modification of chitosan and chitosan derivatives enables an appropriate hydrophilic/hydrophobic balance to promote self-assembly in an aqueous or polar medium. This modification is usually achieved by grafting hydrophobic moieties to the polysaccharide chains. The hydrophobically modified chitosan chains self-aggregate in the hydrophilic media as illustrated in Figure 11. The following sections are shown to illustrate the state of the art of this method of chitosan and chitosan derivatives NPs preparation.

### 3.1. Hydrophobically Modified Chitosan and Chitosan Oligosaccharides

Deoxycholic acid-modified CS self-aggregates have been proposed as a gene delivery system for DNA transfection in cells [145,146]. This system is based on the complex formation between plasmid DNA and positively charged chitosan self-aggregates, which produces micelle-like nanoparticles having controlled dimensions for the effective gene delivery to cells. The hydrophobic modification of chitosan was accomplished with deoxycholic acid that is mediated by carbodiimide coupling (1-ethyl-3-(3-dimethylaminopropyl) carbodiimide, EDC) for amide bond formation. Self-aggregates were obtained by varying the chitosan/deoxycholic acid ratio (degree of substitution of chitosan, DS from 0.02 to 0.1) and the molecular weight of the reacting CS (molecular weight, MW from 5 to 200 kDa). They exhibited hydrodynamic sizes ranging from 132 to 300 nm. For CS molecular weights higher than 40 kDa, a transition from a bamboo-like cylindrical structure to a poorly organized bird nest-like structure of self-aggregates was proposed. The DNA-CS complex formation had strong dependency on the size and structure of CS self-aggregates and significantly influenced gene transfection efficiency (up to a factor of 10) [146].

Similarly, Wang et al. prepared cholesterol-modified chitosan self-aggregates with succinyl linkages mediated by EDC coupling amidation of CS, thus attaining a DS of 0.073 and hydrodynamic diameters of 417.2 nm. Epirubicin was used as a model anticancer drug. It was physically entrapped into the cholesterol-CS self-aggregates, forming almost spherical nanoparticles of 338.2 to 472.9 nm with the epirubicin loading content increasing from ca. 8 to 14%. The controlled release of epirubicin from the loaded nanoparticles was slow, reaching a total release of 24.9% in 48 h [147].

CS-cholesterol self-aggregates were also synthesized with another approach. Prior phthaloylation of CS enabled the esterification of the primary –OH group at C6 with EDC/*N*-Hydroxysuccinimide pre-activated cholesterol succinate. Later, CS deprotection afforded 6-*O*-cholesterol-modified chitosans (DS of 0.017, 0.04, and 0.059) that self-assembled, forming nanoparticles of 100–240 nm sizes. These NPs were capable of physically entrapping the all-trans retinoic acid with different drug loading contents, encapsulation efficiencies, and particle sizes. The sustained release of the all-trans retinoic acid extended over 72 h [148].

Chitosan oligosaccharides (low molecular weight CS produced by depolymerization) are usually preferred over high molecular weight CS for pharmaceutical applications [149]. Chitosans with molecular weights ranging from few hundreds daltons (c.a. trimers, tetramers) to 20 kDa have been referred to as chitosan oligomers [3]. Thus, Hu et al. prepared a CS oligosaccharide (ca. 19 kDa weight average molecular weight) hydrophobically modified with stearic acid and encapsulated paclitaxel or doxorubicin for their controlled delivery [149,150,151,152]. CS oligosaccharide (COS) modification was conducted with stearic acid by an EDC mediated amide linkage reaction, achieving COS substitution degrees of 0.035, 0.05, 0.12, 0.255, and 0.42 [150,151,152,153]. Further glutaraldehyde cross-linking of COS micelle shells before and after paclitaxel physical entrapping enabled drug loading contents of up to 94% and to control the micelle size and paclitaxel release rate [150]. A reduction of micelle diameters from 322.2 to 272.0 nm was observed after glutaraldehyde cross-linking for the blank COS-stearic acid particles and from 355.0 to 305.3 nm for the doxorubicin-loaded COS-stearic acid particles. The Zeta-potential of particles was reduced from +57.1 to +34.2 mV and from +69.1 to +51.8 mV, respectively [150]. Shell cross-linking of doxorubicin-loaded COS-stearic acid micelles also showed enhanced cytotoxicity to A549, LLC, and SKOV3 cancer cell lines [150].

To reduce the observed initial burst release during the dilution of doxorubicin-loaded COS-stearic acid micelles by body fluid, stearic acid was also physically encapsulated into the micelle core [152]. The hydrodynamic diameter of stearic acid-loaded COS-stearic acid micelles increased significantly from 27.4 nm to ca. 60 nm for a 10 wt % of stearic acid/COS-*g*-stearic acid micelles, while Zeta-potential decreased from +51.7 mV to ca. +35 mV [151]. The incorporation of stearic acid that was physically entrapped in the core of doxorubicin-loaded COS-*g*-stearic acid micelle significantly reduced the drug release rate. 

Hu et al. also studied the dual functionalization of COS with stearic acid and doxorubicin cis-aconitate [152]. To this end, a previously prepared COS-stearic acid conjugate (DS in stearic acid of ca. 0.06) was further reacted with doxorubicin cis-aconitate by EDC mediated amidation. This produced COS conjugates with doxorubicin contents of 3, 6, and 10%. DOX-*g*-COS-*g*-stearic acid self-aggregated in an aqueous medium giving micelle sizes of 40.1, 70.7, and 105.8 nm, respectively, and Zeta-potential values of +43.7, +40.2, and +32.0 mV, respectively [152]. 

Chitosan has also been hydrophobically modified with different acyl groups mediated by amide linkage formation with different anhydrides and acyl chlorides such as dl-Lactide (PLA unit modifying the CS), propionic and hexanoic anhydrides, nonaoyl chloride, lauroyl chloride, pentadecanoyl chloride, and stearoyl chloride [153,154]. It was observed that the micelle size of blank CS-PLA increased with an increase of the degree of substitution with PLA units or with the increase of side chain length for the different acyl groups (propionate, hexanoate, nonanoate, etc.). Furthermore, the Zeta-potential changed from +26.0 mV for propionyl chitosan to +10.2 mV for hexanoyl chitosan and remained ca. +13 to +15 mV for the other acyl chitosans. Drug loading content and drug release rate were also influenced by the CS substitution degree or the chain length of the acyl substituents of CS. Rifampin loading content increased and drug release rate decreased with the increase of CS substitution with PLA units [154]. Vitamin C loading content increased and drug release rate decreased with the chain length of the acyl group modifying CS [154].

Water soluble chitosan *N*,*O*6-acetyl chitosan was prepared for future hydrophobic modification with different steroids and dl-α-tocopherol for the sustained release of agrochemicals, testosterone and vitamin E [155]. Drug content achieved values between 11.8 and 56.4 wt %. The CS-steroid and CS-tocopherol micelles formed showed hydrodynamic sizes of ca. 200 to 360 nm in phosphate buffered saline solution (PBS), with Zeta-potential values varying from +7 to +22.7 mV in bi-distilled water. Sustained releases were achieved for the steroids and tocopherol from the CS particles and the biological activity of the released drug appeared unaffected [155].

Amphiphilic block or graft copolymers of phthaloyl chitosan with different materials as poly(ethylene glycol), *N*-vinyl-2-pyrrolidone, and ε-caprolactone have a wide range of pharmaceutical applications [156,157,158,159,160,161,162]. For example, *N*-phthaloylchitosan-*g*-mPEG micelles have been physically loaded with camptothecin and all-trans retinoic acid for their controlled release [156,157,158]. These micelles exerted a protective effect from hydrolysis on the loaded drug (camptothecin, which is sensitive to hydrolysis of the lactone group) or photodegradation (all-trans retinoic acid). Furthermore, a continuous release without an initial burst of prednisone acetate from *N*-phthaloylchitosan-*g*-polyvinylpyrrolidone micelles was achieved [159]. 

There are also several reports showing that chitosan-*graft*-polycaprolactone nanomicelles have been physically loaded with 7-ethyl-10-hydroxy-camptothecin, BSA, paclitaxel, and 5-fluorouracil [160,161,162,163].

Another amphiphilic copolymer of CS was synthesized from *N*-acetyl histidine as the hydrophobic segment and arginine-grafted chitosan by EDC carbodiimide-mediated coupling for the controlled delivery of doxorubicin [164]. The key finding was the effectiveness of doxorubicin loaded *N*-acetyl histidine and arginine-grafted CS for the suppression of both the sensitive and resistant human breast tumor cell line (MCF-7) in a dose- and time-dependent pattern. 

More details of prepared hydrophobically modified CS and CS oligosaccharide conjugates can be found in Table 4.

### 3.2. Hydrophobically Modified Glycol Chitosan

**Figure 12:**
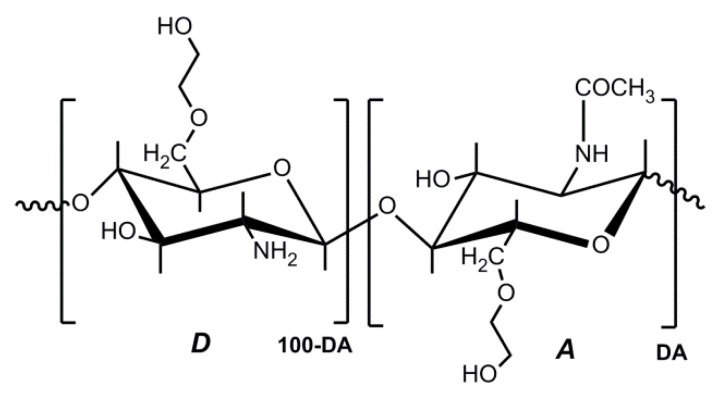
The chemical structure of glycol chitosan.

The limited water solubility of CS and the precipitation of some self-aggregated chitosan conjugates restricts its application in medical practice as a drug delivery system. In contrast, glycol chitosan (GC) exhibits good water solubility at all pHs, good biocompatibility, and is widely applied as a hydrophobic drug and gene carrier [165,166,167,168,169,170]. The structural units of GC are shown in Figure 12.

GC has been functionalized with cholanic acid, cholesterol, deoxycholic acid, vitamins, testosterone, doxorubicin, and other hydrophobic compounds using mostly an EDC-mediated coupling reaction to achieve the amidation of CS amine groups with the desired carboxylic acid or acyl chloride of the hydrophobic substituent. Further physical encapsulation of anticancer drugs or bioactive compounds in the core of self-assembled GC hydrophobically-modified micelles is usually performed.

Hwang et al. introduced cholanic acid in GC. The resulting GC-cholanic acid micelles can be easily loaded with the anticancer drug docetaxel [165]. Docetaxel loaded GC-cholanic acid synthesized spontaneously and was self-assembled as 350 nm aggregates in an aqueous medium. During in vivo experiments in mice, these docetaxel loaded nanoaggregates showed higher anticancer efficacy to A549 lung cancer cells and reduced toxicity when compared to the free drug.

The anticancer drug camptothecin has also been encapsulated into self-aggregates of GC-cholanic acid, with a drug loading efficiency of above 80% [166]. GC-cholanic acid micelles protected the lactone ring of camptothecin from hydrolysis and camptothecin loaded micelles showed significant antitumor activity towards MDA-MB231 human breast cancer cells that were implanted in nude mice. The 5β-cholanic hydrophobic functionalization of both GC and polyethylenimine and later mixing of both modified polymers, made it possible to obtain self-assembled nanoparticles of ca. 350 nm with a Zeta-potential of +23.8 mV, for the delivery of siRNA in tumor-bearing mice [167]. The siRNA-GC-polyethylenimine complex transfected the B16F10 tumor cells, efficiently inhibiting the RFP gene expression of RFP/B16F10-bearing mice. Thus, GC-polyethylenimine self-aggregates were revealed as promising gene carrier for cancer treatment [167]. GC-cholanic acid self-aggregates have also been proposed for the delivery of RGD peptide and indomethacin [168,169].

The hydrophobic modification of GC with deoxycholic acid and the later physical encapsulation of palmityl-acylated exendin-4 peptide in formed self-assembled nanogels for a long-acting anti-diabetic inhalation system was studied by Lee et al. [170]. Results were promising, with the ca. 72 h residence of the administered anti-diabetic drug (palmityl-acylated exendin-4 peptide) in the lungs, good hypoglycemic response, and acceptable toxicity.

In another approach, the hydrophobic modification of GC with the drug to be delivered has been explored. Quiñones et al. synthesized GC hydrophobically-modified with ergocalciferol hemisuccinate, tocopherol hemiesters, and testosterone 17β-hemisuccinate for the controlled release of vitamin D2, vitamin E and testosterone [171,172,173]. The degrees of substitution of GC with the vitamins and the testosterone reached values of 0.039 for vitamin D2, 0.21 to 0.36 for vitamin E and 0.015 for testosterone. The GC-vitamin and GC-testosterone conjugates formed self-assembled NPs in an aqueous medium with hydrodynamic sizes from 280 to 500 nm andZeta-potential values of +7.7 to +36.5 mV. The sustained release of covalently linked vitamins and testosterone from the GC self-aggregates was observed in an acidic medium for 3 to 4 days. 

The hydrophobic modification of GC with an *N,N*-diethylnicotinamide-based oligomer enabled a high paclitaxel loading content with an EE of up to 98% [174]. The hydrodynamic diameter of the blank hydrophobically modified GC was 313 ± 20 nm in PBS. Paclitaxel loaded modified GC particles with a drug loading content of 9.8, 18.9, and 23.9 wt % exhibited hydrodynamic sizes of 331 ± 25 nm, 354 ± 23 nm, and 363 ± 32 nm, respectively. A sustained release of paclitaxel from the GC self-aggregates was observed. Overall, the anticancer assessment of the paclitaxel loaded GC particles appears promising in cancer therapy.

Doxorubicin encapsulation in GC-3-diethylaminopropyl self-aggregates and the hydrophobic functionalization of GC with doxorubicin was also accomplished for the evaluation of doxorubicin delivery systems for cancer therapy [175,176]. The hydrodynamic parameters of GC-based self-aggregates discussed are summarized in the Table 5.

### 3.3. Hydrophobically Modified Carboxymethyl Chitosan

*O*-Carboxymethyl chitosan, typically named carboxymethyl chitosan (CMCS), has been hydrophobically modified with oleoyl chloride in pyridine/dichloromethane or with linoleic acid using an EDC-mediated amide linkage reaction [177,178,179].

Oleoyl-modified CMCS formed self-aggregates in an aqueous medium with average hydrodynamic diameters that depended on the molecular weight of the chitosan used to prepare the CMCS [177,178]. Hydrodynamic diameters of 157.4 nm (CS with molecular weight of 50 kDa), 161.8 nm (CS with molecular weight of 38 kDa), 274.1 nm (CS with molecular weight of 170 kDa), and 396.7 nm (CS with molecular weight of 820 kDa) have been reported for different oleoyl-modified CMCS. The Zeta-potential values observed for blank oleoyl-modified CMCS particles were +16 ± 1 mV, +17.2 ± 0.9 mV, and +20 ± 1 mV. Rifampicin and microbial antigens were physically entrapped in the oleoyl-modified CMCS micelles with drug loading efficiency of 20% for rifampicin and ca. 52 to 62.5% for microbial antigens. The sustained release of encapsulated drugs was extended until 40–48 h [177,178]. 

Linoleic acid modified CMCS self-aggregated micelles were loaded with the anticancer drug, adriamycin, for a sustained release [179]. The average hydrodynamic diameter of the blank linoleic-modified CMCS was 418 ± 18 nm. Adriamycin was slowly released from the micelles for about three days. Results are summarized in Table 6.

### 3.4. Hydrophobically Modified Succinyl Chitosan

**Figure 13:**
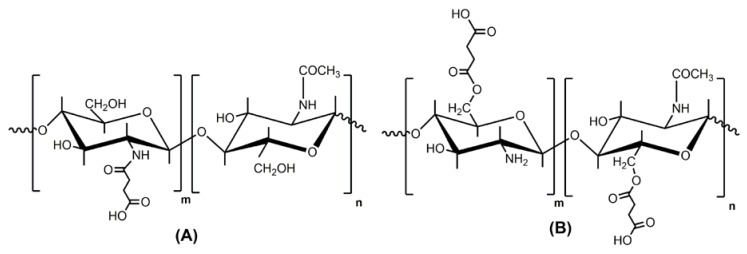
The chemical structure of *N*-succinyl chitosan (**A**) and O6-succinyl chitosan (**B**).

Water soluble succinyl chitosans have been prepared by amidation (*N*-succinyl chitosan) and esterification (*O*6-succinyl chitosan) of chitosan by its reaction with succinic anhydride (Figure 13).

Xiangyang et al. reported the preparation of *N*-succinyl-*N’*-octyl chitosan micelles as doxorubicin carriers for an effective anti-tumor activity [180]. Average hydrodynamic sizes of doxorubicin loaded modified succinyl chitosan (SCS), which depended on the amount of octyl chains and the drug loading content, was between 100 to 200 nm. Doxorubicin loaded SCS particles showed a sustained release and more cytotoxic activity against HepG2, A549, BGC, and K562 cancer cell lines than parent doxorubicin.

In another study on SCS, the interactions between the polymer and BSA in the nanoaggregates are inspected using different techniques [181]. The authors concluded that no significant change on the conformation of BSA occurred during chain entanglements between the protein and the *N*-succinyl chitosan. The hydrodynamic sizes of the micelles formed are reported in Table 7.

The synthesis of *O*6-succinyl chitosan involves phthaloyl protection of chitosan, the reaction with succinic anhydride, and deprotection (removal of the phthaloyl groups). Further hydrophobic modification of free amine groups of *O*6-succinyl chitosan with tocopherol succinate mediated by an EDC activated coupling reaction, made it possible to prepare cationic self-assembled SCS nanoparticles with hydrodynamic diameters of 254 ± 4 nm and Zeta-potential values of +36.3 ± 0.9 mV [172]. The sustained release of covalently linked vitamin E (tocopherol) was extended up to 96 h. The results are shown in Table 7.

### 3.5. Hydrophobically Modified Trimethyl Chitosan

*N*,*N*,*N*-Trimethyl chitosan (TMC) is a water soluble derivative of chitosan that is prepared by exhaustive *N*-methylation of some free amine groups of CS using iodomethane.

TMC has been hydrophobically modified with octyl, decanoyl, lauryl, lactose, and palmitoyl substituents for hydroxycamptothecin and harmine encapsulation in the hydrophobic core [182,183,184,185]. *N*-octyl-*N*-trimethyl chitosan and *N*-lauryl-*N*-trimethyl chitosan were self-assembled in an aqueous medium as micelles of 23.5 and 20.8 nm, while *N*-decanyl-*N*-trimethyl chitosan formed micelles with a hydrodynamic diameter of 277.2 nm.

Hydroxycamptothecin loaded *N*-alkyl-*N*-trimethyl chitosan micelles showed a sustained release of the anticancer drug, with improved pharmacokinetic properties and the stability of the camptothecin lactone ring in vivo [182]. On the other hand, the harmine loaded hydrophobically modified TMC released 65.3% of the encapsulated drug in three days at pH 7.4 [183,184].

Mi et al. investigated the preparation of self-assembled NPs by TMC and poly(γ-glutamic acid) for the oral delivery of insulin [185]. The hydrodynamic diameters and Zeta-potential values of blank and insulin loaded TMC/poly(γ-glutamic acid) NPs are presented in Table 8.

### 3.6. Other Hydrophobically Modified Chitosan Derivatives

*N*-octyl-*O*-sulfate chitosan (NOSC) micelles have been prepared from chitosan for the sustained release of physically entrapped paclitaxel for cancer therapy [186,187,188]. Paclitaxel loaded *N*-octyl-*O*-sulfate chitosan micelles showed hydrodynamic diameters of ca. 200 nm and Zeta-potential values of ca. −30 mV [186,187]. On the other hand, the additional modification of *N*-octyl-*O*-sulfate chitosan with polyethylene glycol monomethyl ether reduced the hydrodynamic sizes of the paclitaxel loaded NOSC to ca. 100 nm [188]. The anticancer drug loaded NPs exhibited reduced toxicity and improved the bioavailability of encapsulated paclitaxel [186,187,188].

Pedro et al. synthesized *N*-dodecyl-*N*′-glycidyl(chitosan) for the delivery of quercetin [189]. The hydrodynamic parameters of quercetin loaded hydrophobically modified CS micelles were measured by dynamic light scattering showing sizes from 140 to 260 nm and Zeta-potential values from +18.7 to +30.4 mV at pH 7.4. At pH 5.0 the sizes ranged from 150 to 300 nm and the Zeta-potential values varied from +14.1 to +29.9 mV, showing the dependence of both the parameters on sample concentration at both pHs. pH was also found to play a key role on quercetin release from the micelles. The results are summarized in Table 9.

## 4. Conclusions

A considerable amount of research is going on for the self-assembling preparation of chitosan nanoparticles in drug delivery applications. In particular, the nanoparticle preparations by polyelectrolyte complexation and by the self-assembly of hydrophobically modified chitosans are able to encapsulate the drug under mild conditions without losing their stability and biocompatibility. Therefore, chitosan based self-assembled nanoparticles have great potential, as well as multiple applications for the future in the design of novel drug delivery systems.

## Figures and Tables

**Table 1:** Chitosan-Alginate PEC nanoparticles. The intervals shown generally indicate extreme values obtained under different preparation conditions.

Procedure	Active agent	Particle size (nm)	Zeta-potential (mV)	Ref.
*Complex coacervation*				
CS added into ALG CS into ALG-DOX	Ibuprofen Dipyridamole	320 to 700 ^b^	+6.34 ^b^ to –44.5 ^b,^*	[67]
	Gatifloxacin ^a^	347 ^c^	+38.6 ^c^	[73]
	Doxorubicin	100 ± 28 ^b^	+36 ± 3 ^b^	[68]
		100 ± 35 ^c^	+35 ± 4 ^c^	
ALG added into CS ALG into Thiolated CS	Amoxicillin ^a^	264 to >601	+ 35 to + 61.9	[69]
	Fluorescein	338 ± 16 ^b^	+34 ± 8 ^b^	[74]
	isothiocyanate	266 ± 7 ^c^	+30 ± 4 ^c^	
	Fluorescein	338 ± 16 ^b^	+34 ± 8 ^b^	
	isothiocyanate	266 ± 7 ^c^	+30 ± 4 ^c^	
ALG + TPP added into CS	Insulin	260–525	+41 to +50	[70]
*Ionotropic pregelation of alginate plus PEC coating with CS*				
CS into Ca/(ALG + drug)	Insulin	781 ± 61 ^b^	−15 ± 2 ^b^	[75]
		748 ± 217	−6 ± 2 ^c^	
	Vitamin-B2	120 ± 50 ^b^	−30.9 ± 0.5 ^b^	[71]
		104 ± 67 ^c^	−29.6 ± 0.1 ^c^	
	Acetamiprid	201.5	−32.1	[76]
CS + EGF into Ca/ALG	EGF-antisense ^a^	194–1435	~+30	[77]
CS + plasmid into Ca/ALG	pEGFP plasmid	161	+29.3	[78]
*o/w ALG microemulsion followed by ionotropic gelation and further complexation with CS*				
	Turmeric oil	522–667	−21.8 to −22.2	[79]
	A.A.	400		[80]
	CDD	410 ± 20	22 ± 1	[72]
LMWAlg + OligoCS	BSA	134–229		[81]

^a^ Optimization performed; ^b^ unloaded particle; ^c^ loaded particle; A.A., aminoacid derivatives; CDD, curcumin diethyl disuccinate; * pH 3.0.

**Table 2:** Chitosan-Pectin PEC nanoparticles. The intervals shown generally indicate extreme values obtained under different preparation conditions.

Procedure	Active agent	Particle size (nm)	Zeta-potential (mV)	Ref.
*Complex coacervation*				
Pectin added into CS	Insulin	441 ± 32 ^a^		[86]
		580–896 ^b^	+62 ± 3 ^b^	
		* 650 ± 86 ^b^	+33 ± 4 ^b^	
	Curcumin	10–59 (dry NPs)		[87]
	Insulin	1175–2618 ^a^	−22.5 to +35.0 ^a^	[23]
		964–2510 ^b^	−22.4 to +33.2 ^b^	
	Nisin	301–712 ^b^		[88]
	None	560–1000	+20 to +26	[84]
CS added into Pectin	None	460–1110	+19 to +28	[84]
*Combined ionotropic gelation and complex coacervation*				
Pectin + TPP added into CS	Insulin	375–7239	+10.6 to +32.7	[86]
CS added into Pectin + TPP	OVA	250–750 ^a^	−20 to −29 ^a^	[85]
CS + TPP added into Pectin	BSA	200–400 ^a^	−15 to −45 ^a^	[85]
		700–1250 ^b^	−38 ^b^	
*Ionotropic pregelation of pectin plus PEC coating with CS*				
CS added into Pectin + CaCl_2_	OVA	419 ^a^	−30.4 ^a^	[84]
		302–409 ^b^	−21.9 to −26.0 ^b^	

^a^ Unloaded particle; ^b^ loaded particle * The CS solution contained Ca^2+^ ions.

**Table 3:** Chitosan-Dextran sulfate PEC nanoparticles. The intervals shown generally indicate extreme values obtained under different preparation conditions.

Procedure	Active agent	Particle size (nm)	Zeta-potential (mV)	Ref.
*Complex coacervation*				
DS added into CS		>244 ^a^	−47.1 to −60 ^a^	[99]
	BSA	478–1138 ^b^	−28.0 to +56.4 ^b^	
	Rhodamine 6G	245–3521 ^b^	−31.0 to +34.0 ^b^	
CS added into DS	Insulin	489–665 ^b^	−0.4 to −21.5 ^b^	[100]
		527–1577 ^b^	−20.6 to +11.5 ^b^	[101]
	Amphotericin B	616–891 ^a^		[93]
		644–1040 ^b^	−27 to −37	
	REPIFERMIN^®^	239	−18.4	[94]
		306	−15.5	
Mixing with agitation	Hydralazine	290 ± 60 ^a^	−7 ± 4 ^a^	[102]
		340 ± 50 ^b^	−5 ± 1 ^b^	

^a^ Unloaded particle; ^b^ loaded particle.

**Table 4:** Hydrophobically modified chitosan and chitosan oligosaccharides.

Hydrophobic moiety	Active agent	Particle size (nm)	Zeta-potential (mV)	Ref.
deoxycholic acid	DNA	162 ± 18 ^a^		[145]
		~300 ^b^		
		130–300 ^a^		[146]
cholesterol	Epirubicin	417 ± 18 ^a^		[147]
		338–473 ^b^		
6-*O*-cholesterol	All-trans retinoic acid	100–240 ^a^	+24.5 to +25.9 ^a^	[148]
		192–222 ^b^		
stearyl	Paclitaxel	28.1–74.6 ^a^	+39.0 to +53.2 ^a^	[149]
		35.8–175.1 ^b^	+44.0 to +58.7 ^b^	
	Doxorubicin	272–322 ^a^	+34.2 to +57.1 ^a^	[150]
		305–355 ^b^	+51.8 to +69.1 ^b^	
		27.4 ± 2.4 ^a^	+52 ± 3 ^a^	[151]
		20.4 ± 1.1 ^b^	+53.1 ± 14.4 ^b^	
stearyl + doxorubicin	Doxorubicin	40.1–105.8 ^b^	+32.0 to +43.7 ^b^	[152]
Acyl	Rifampin	154–181 ^a^		[153]
		163–210 ^b^		
	Vitamin C	444–487 ^a^	+10.2 to +28.9 ^a^	[154]
		216–288 ^b^	+5.9 to +18.4 ^b^	
*N,O*6-acetyl + steroid	Steroids	197–358 ^b^	+7 to +22.7 ^b^	[155]
*N,O*6-acetyl + tocopherol	Vitamin E	275 ± 5 ^b^	+14.9 ± 0.7 ^b^	
phthaloyl	Camptothecin	~170 ^a^		[156]
		~200–267 ^b^		
		~50–100 ^a^		
		~100–250 ^b^		[157]
	All-trans retinoic acidPrednisone acetate	~50–100 ^a^		[158,159]
		~80–160 ^b^		
		89.8 ^a^		
		143.3 ^b^		
polycaprolactone, (Chitosan-grafted)	7-Ethyl-10-hydroxy-camptothecin	47–113 ^a^	+26.7 to +50.8 ^a^	[160]
		63–152 ^b^	+25.6 to +48.8 ^b^	
	BSA	168.44 ^b^		[161]
		200.7 ^b^		
		435 ± 25 ^a^		
	Paclitaxel	408–529 ^b^	+27.5 ± 1.1 ^a^	[162]
		61.4–108.6 ^a^	+30.9 to +33.3 ^b^	
	5-Fluorouracil	67.9–96.7 ^b^	+18.9 to +43.1 ^b^	[163]
*N*-acetyl histidine	Doxorubicin	218 ^a^	+40.1 ± 2.8 ^a^	[164]
		185.3–218.3 ^b^	+36.3 to +40.1 ^b^	

^a^ Unloaded particle; ^b^ loaded particle; * 5 mg/mL.

**Table 5:** Hydrophobically modified glycol chitosan.

Hydrophobic moiety	Active agent	Particle size (nm)	Zeta-potential (mV)	Ref.
Cholanic acid	Docetaxel	350 ^b^	+23.8 ± 0.9 ^a^ +10.0 ± 0.8 ^b^	[165]
	Camptothecin	254 ^a^		[166]
		279–328 ^b^		
	siRNA	350 ^a^		[167]
		250 ^b^		
	RGD peptide	224 ^a^		[168]
		189–265 ^b^		
Cholesterol	Indomethacin	228 ^a^		[169]
		275–384 ^b^		
Deoxycholic acid	Palmityl-acylated exendin-4	~52–250 ^a^		[170]
Ergocalciferol	Vitamin D2	279 ± 7 (PBS)	+7.7 ± 0.1	[171]
dl-α-tocopherol	Vitamin E	284–496 (PBS)	+11.7 to +36.5	[172]
Testosterone	Testosterone	332 ± 4 (PBS)	+9.7 ± 0.6	[173]
*N,N*-diethylnicotinamide-based oligomer	Paclitaxel	313 ± 20 ^a^		[174]
		331–363 ^b^		
3-Diethylaminopropyl	Doxorubicin	102 ^a^	−0.9 ^a^	[175]
Doxorubicin	Doxorubicin	238 ^a^ 342 ^b^		[176]

^a^ Unloaded particle; ^b^ loaded particle.

**Table 6:** Hydrophobically modified carboxymethyl chitosan.

Hydrophobic moiety	Active agent	Particle size (nm)	Zeta-potential (mV)	Ref.
Oleoyl	Rifampicin	161.8 ^a^		[177]
	Microbial	157.4–396.7 ^a^	+15.6 to +19.6 ^a^	[178]
	antigen	237.6–482.3 ^b^	+14.2 to +17.1 ^b^	
		331.6–573.9 ^b^	+12.8 to +16.3 ^b^	
Acyl	Adriamycin	418 ± 18 ^a^		[179]

^a^ Unloaded particle; ^b^ loaded particle.

**Table 7:** Hydrophobically modified succinyl chitosan.

Hydrophobic Moiety	Active Agent	Particle Size (nm)	Zeta-Potential (mV)	Ref.
Octyl	Doxorubicin	130.4–150.1 ^a^		[180]
		155.4–170.1 ^b^		
Acyl	BSA	~50–100 ^a^		[181]
		~100–200 ^b^		
dl-α-tocopherol	Vitamin E	254 ± 4	+36.3 ± 0.9	[172]

^a^ Unloaded particle; ^b^ loaded particle.

**Table 8:** Hydrophobically modified trimethyl chitosan.

Hydrophobic Moiety	Active Agent	Particle Size (nm)	Zeta-Potential (mV)	Ref.
Alkyl	Hydroxy-camptothecin	20.8–277.2 ^a^		[182]
		26.0–273.1 ^b^		
Palmitoyl	Harmine	193.4 ± 3.1 ^b^	+26.67 ^b^	[183,184]
Acyl	Peptide drugs	101.3–106.3 ^a^	+30.6 to +36.2 ^a^	[185]
		522 ± 6 ^b,^*	+14.2 ± 0.6 ^b,^*	

^a^ Unloaded particle; ^b^ loaded particle; * pH 7.4.

**Table 9:** Other chitosan derivatives.

Hydrophobic Moiety	Active Agent	Particle Size (nm)	Zeta-Potential (mV)	Ref.
Octyl	Paclitaxel	~200 ^b^		[186]
		200.8 ^b^	−31.1 ^a^ −28.8 ^b^	[187]
		104.3–133.4 ^b^		[188]
Acyl	Quercetin	140–300 ^a^	+14.1 to +30.4 ^a^	[189]

^a^ Unloaded particle; ^b^ loaded particle.
